# TAZ deficiency exacerbates psoriatic pathogenesis by increasing the histamine-releasing factor

**DOI:** 10.1186/s13578-024-01246-0

**Published:** 2024-05-11

**Authors:** Jiseo Song, Hyo Kyeong Kim, Hyunsoo Cho, Suh Jin Yoon, Jihae Lim, Kyunglim Lee, Eun Sook Hwang

**Affiliations:** College of Pharmacy and Graduate School of Pharmaceutical Sciences, Ewha Womans University, Seoul, 03760 Korea

**Keywords:** HRF, IMQ, Mast cell, Protein degradation, TAZ

## Abstract

**Background:**

Transcriptional coactivator with PDZ-biding motif (TAZ) is widely expressed in most tissues and interacts with several transcription factors to regulate cell proliferation, differentiation, and death, thereby influencing organ development and size control. However, very little is known about the function of TAZ in the immune system and its association with inflammatory skin diseases, so we investigated the role of TAZ in the pathogenesis of psoriasis.

**Results:**

Interestingly, TAZ was expressed in mast cells associated, particularly in lysosomes, and co-localized with histamine-releasing factor (HRF). TAZ deficiency promoted mast cell maturation and increased HRF expression and secretion by mast cells. The upregulation of HRF in TAZ deficiency was not due to increased transcription but to protein stabilization, and TAZ restoration into TAZ-deficient cells reduced HRF protein. Interestingly, imiquimod (IMQ)-induced psoriasis, in which HRF serves as a major pro-inflammatory factor, was more severe in TAZ KO mice than in WT control. HRF expression and secretion were increased by IMQ treatment and were more pronounced in TAZ KO mice treated with IMQ.

**Conclusions:**

Thus, as HRF expression was stabilized in TAZ KO mice, psoriatic pathogenesis progressed more rapidly, indicating that TAZ plays an important role in preventing psoriasis by regulating HRF protein stability.

**Graphical Abstract:**

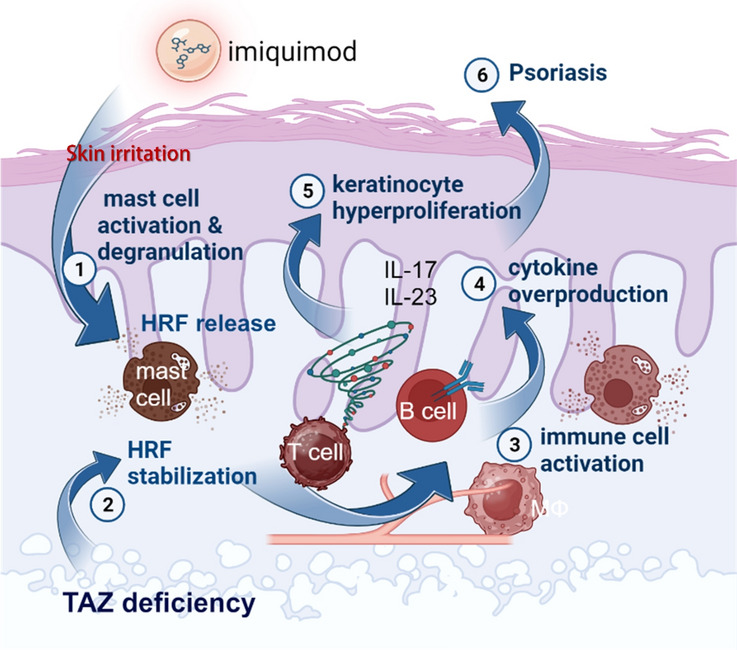

## Highlights

- HRF is expressed in lysosomes and associated with TAZ expression.
- HRF expression is increased in TAZ-deficient immune cells.
- HRF protein stability is controlled by TAZ, resulting in stabilized HRF in TAZ deficiency.
- TAZ deficiency promotes susceptibility to IMQ-induced psoriasis, leading to exacerbated psoriatic inflammation.

## Background

Transcriptional coactivator with PDZ-biding motif (TAZ) is a transcriptional coregulator that is expressed in most cell and tissue types and, like yes-associated protein (YAP), regulates organ development and size through the mediation of the Hippo signaling pathway [1, 2]. TAZ interacts with various transcription factors through multiple protein–protein interaction domains and modulates gene expression in a context-dependent manner [3]. For example, TAZ is expressed in mesenchymal stem cells and regulates their differentiation into osteoblasts, adipocytes, and muscle cells through interaction with RUNX2, PPARγ, and MyoD, respectively, which contributes to the regeneration and repair of musculoskeletal tissues [4–6]. TAZ is essential for myogenic differentiation and injury-induced liver regeneration and protects against liver fibrosis [7–9]. Furthermore, TAZ promotes insulin production by binding to PDX1 in pancreatic islet cells and activates mitochondrial biogenesis in muscle tissue, thus improving glucose sensitivity [10–13]. Therefore, TAZ deficiency causes functional defects in the lungs, kidneys, and reproductive system and increases the risk of developing metabolic diseases such as osteoporosis, obesity, and diabetes [12, 14–16]. Although TAZ has been extensively studied in various organs and tissues, whether and how it affects the immune system remains relatively unclear. It has been reported that TAZ deficiency promotes the differentiation of regulatory T (Treg) cells from CD4 T cells but suppresses the differentiation of T helper 17 (Th17) cells, thereby attenuating Th17-mediated autoimmune diseases [17]. In addition, TAZ is expressed in macrophages, and its deficiency alters the maturation and differentiation of pro-inflammatory macrophages, attenuating inflammatory responses after myocardial infarction [18]. Thus, TAZ may induce pro-inflammatory responses by promoting inflammatory Th17 cell development and M1 macrophage polarization. However, it is still unclear whether TAZ affects the activity and function of other immune cells, including mast cells, and whether and how TAZ contributes to the development of inflammatory skin diseases, such as psoriasis.

Psoriasis is a systemic inflammatory skin disease characterized by thick, scaly plaques on the skin due to dysregulated immune responses and increased infiltration of various innate and adaptive immune cells, such as dendritic cells, macrophages, Th1, and Th17 cells [19–22]. Innovative single-cell RNA sequencing technologies have identified the enrichment of myeloid dendritic cells and reprogrammed macrophages in the psoriatic lesional skin of patients [23, 24]. In particular, the infiltration of M1 macrophages, Th1 cells, and Th17 cells leads to the massive production of various inflammatory cytokines, including TNF-α, IFN-γ, IL-17, and IL-23, exacerbating the pathogenesis of psoriatic skin inflammation in imiquimod (IMQ) animal models and humans, despite the anti-inflammatory function of Treg cells [19, 25, 26]. Recently, non-exhausted CD8 + T cells have also been identified as cells that infiltrate the psoriatic skin [27]. Most notably, mast cells, which are highly concentrated in the skin, are crucial in initiating and regulating innate and adaptive immune responses by communicating with other cells [28]. Mast cells are first activated by external stimuli or pathogen invasion and subsequently release various inflammatory mediators, such as cytokines, IgE-dependent histamine-releasing factor (HRF, also known as translationally controlled tumor protein), histamine, proteases, and prostaglandins. This process exerts immunomodulatory functions or triggers allergic responses [29, 30].

Interestingly, YAP, which exhibits similar functions to TAZ, has been reported to be upregulated in skin epithelial cells of psoriasis, and reduction of YAP expression ameliorates psoriasis [31, 32]. Therefore, based on the pro-inflammatory activity of TAZ through the regulation of macrophages and T cells and its functional similarity to YAP, we sought to determine whether TAZ contributes to the exacerbation of psoriasis pathogenesis and to analyze its function in the immune response by mast cells.

## Methods

### Materials

Compound 48/80 (C48/80, C2313), bafilomycin A1, 4′,6-diamidino-2-phenyl-indole (DAPI, D9542), Giemsa stain (51811-82-6), and toluidine blue O (T3260) reagents were purchased from Sigma-Aldrich (St. Louis, MO, USA). DMEM (12100-061) and FBS (16140-071) for cell culture were purchased from Thermo Fisher Scientific (Waltham, MA, USA).

### Generation of BMMCs

Bone marrow cells were isolated from the femurs and tibias of WT and TAZ KO mice (male, 8–10 weeks old) and cultured in RPMI-1640 supplemented with recombinant murine IL-3 (rmIL-3, 10 ng/mL, #575502, BioLegend, San Diego, CA, USA) for 28–35 days [33]. BMMCs were used for all functional assays between days 29 and 35 post-isolation. The purity of BMMCs was analyzed by staining with antibodies against FcεRI (#12-5898-82, eBioscience, San Diego, CA, USA) and c-kit (#11-1171-82, eBioscience) and flow cytometry. Cells were acquired on a FACS Calibur flow cytometer (BD Biosciences, San Jose, CA, USA) equipped at the Ewha Fluorescence Core Imaging Center by collecting 100,000 cell-gated events and quantitatively analyzed using the CellQuest software.

### Immunofluorescence staining

BMMCs were treated with or without LPS (1 µg/mL) for 24 h and treated with either vehicle or C48/80 (50 µg/mL) for 5 min before harvest. The cells were fixed with 4% paraformaldehyde and incubated with antibodies against LAMP1 (sc-19992, Santa Cruz Biotec), TAZ (#72804, Cell signaling technology, Danvers, MA, USA; or #560235, BD Bioscience), and HRF (AB37506, Abcam, Cambridge, MA, USA), followed by staining with Alexa Fluor 488- or Alexa Fluor 555-conjugated secondary antibodies (Invitrogen). Cell nuclei were stained with DAPI (1 µg/mL). Cells were observed using a confocal laser scanning microscope (ZEISS LSM 880 with Airyscan, Oberkochen, Germany).

### Giemsa and toluidine blue staining

WT and TAZ KO BMMCs were cultured and harvested for staining with Giemsa or toluidine blue (Diff Quik staining kit, Sysmex Co., Kobe, Japan). The cells were smeared on a slide glass with Cytospin 4, fixed with pure methanol, and stained with 5% Giemsa staining solution. For toluidine blue staining, the cells were fixed with 3% glutaraldehyde and permeabilized with 0.5% Triton X-100, followed by toluidine blue staining. The cells were washed and observed under a microscope.

### β-hexosaminidase assay

WT and TAZ KO BMMCs were cultured and incubated with vehicle or bafilomycin A1 (100 nM) for 24 h. Cell pellets were resuspended in extracellular buffer (10 mM HEPES, 137 mM NaCl, 2.7 mM KCl, 0.4 mM Na_2_HPO_4_.7H_2_O, 1.4 mM CaCl_2_, 1 mM MgCl_2_, 5.6 mM glucose, and 0.04% BSA) and left untreated or treated with 50 μg/mL C48/80 for 5 min. The cell supernatant was harvested and subjected to β-hexosaminidase release assay using 4-nitrophenyl 2-acetamido-2-deoxy-β-D-glucopyranoside in 0.1 M citrate buffer (pH 4.2) (PNAG, 3.4 mg/mL, TCI Chemicals, Japan), followed by measurement at 405 nm. The total amount of β-hexosaminidase was also measured in the cell lysates.

### Mouse model of psoriasis

All animal experiments were approved by the Institutional Animal Care and Use Committee of Ewha Womans University (IACUC 20–040) and conducted in accordance with international guidelines. TAZ KO mice were generated by a strategy of deleting the second exon and homozygous KO males were crossed with heterozygous females to ensure sufficient numbers of KO mice [14, 16]. WT and TAZ KO mice (male, 8‒10 weeks old, 22‒25 g) were divided into the vehicle- and IMQ-treated groups and shaved on the back, followed by daily topical treatment with 62.5 mg of 5% IMQ (Aldara cream, 3 M Pharmaceuticals) or control cream [34]. IMQ was also applied daily to the right ear pinna of the mice for 7 consecutive days, as previously reported [35]. A cumulative score of the Psoriasis Area and Severity Index (PASI) was assessed based on the intensity of redness, thickness, and scaling of the dorsal skin (0: none, 1: mild, 2: moderate, 3: severe). Skin thickness was measured using a dial thickness gauge (Peacock G1-A, Ozaki MFG. Co., Ltd., Tokyo, Japan).

### Histological analysis

The dorsal skin, ear skin, and lymph nodes were collected from WT and TAZ KO mice. The tissues were fixed, embedded, and sectioned at 4 μm thickness. Skin section slides were stained with either hematoxylin and eosin (H&E), trichrome, or toluidine blue, followed by microscopic observation. For immunohistochemistry, skin and lymph node sections were fixed and incubated with antibodies against HRF (ab37506, Abcam), followed by incubation with DAPI (1 μg/mL). Images were viewed under a fluorescence microscope (Axio Observer 7, Carl Zeiss Jena, Germany) at the Ewha Drug Development Research Core Center.

### Immunoblot analysis

Protein samples were collected from cultured cells and tissues (spleen, lymph nodes, and back skin) and extracted using lysis buffer. Serum samples were also collected from the blood of WT and TAZ KO mice and pre-treated to remove serum albumin proteins. Protein extracts and serum samples were analyzed by SDS-PAGE and electrotransfer, followed by incubation with antibodies against HRF (AB37506, Abcam) and TAZ (#8418, YAP/TAZ, Cell signaling technology). A similar amount of protein loading was confirmed by immunoblotting of β-actin (BS-0061R, Bioss, Woburn, MA, USA) and by staining Ponceau S (Sigma). The protein band signal was quantitated using Image J software.

### Human gene transcript analysis

Human gene transcripts in healthy and psoriatic skin were collected from the Gene Expression Omnibus website (https://www.ncbi.nlm.nih.gov/geo/geo2r/?acc=GSE13355) [36]. The expression levels of TAZ and HRF and their correlation were determined by plotting normalized expression values in the skin of healthy subjects and lesional skin of patients with psoriasis using GraphPad Prism.

### Quantitative real-time PCR

Total RNA was isolated from the spleen and lymph nodes of WT and TAZ KO mice or cultured MEFs or spleen cells using TRIzol reagent (Thermo Fischer Scientific) and subjected to reverse transcription, as previously described [12]. Quantitative real-time PCR analysis was performed using a THUNDERBIRD SYBR qPCR mix (TOYOBO, Osaka, Japan) in a Step One Plus real-time PCR system (Applied Biosystems, Carlsbad, CA, USA). The relative HRF transcript levels were calculated after normalization with the Ct values of the β-actin gene. The following specific primer sets were used: *actin*, 5′-caccctgtgctgctcaccgag-3′ and 5′-accgctcgttgccaatagtga-3′ and *HRF*, 5′-accgaaagcacagtagtcacc-3′ and 5′-agtcacaccatcttcacgg-3′.

### Data and statistical analysis

All experiments were performed at least 3 times, and the results were analyzed using GraphPad Prism 7 (GraphPad Software, San Diego, CA, USA). All data are expressed as the mean ± SEM. Statistical significance was determined using one-way ANOVA with post-hoc Tukey′s HSD test or two-tailed Student′s *t*-test. P values less than 0.05 were used to denote a statistically significant difference.

## Results

### TAZ deficiency enhances mast cell maturation and degranulation

**Fig. 1:**
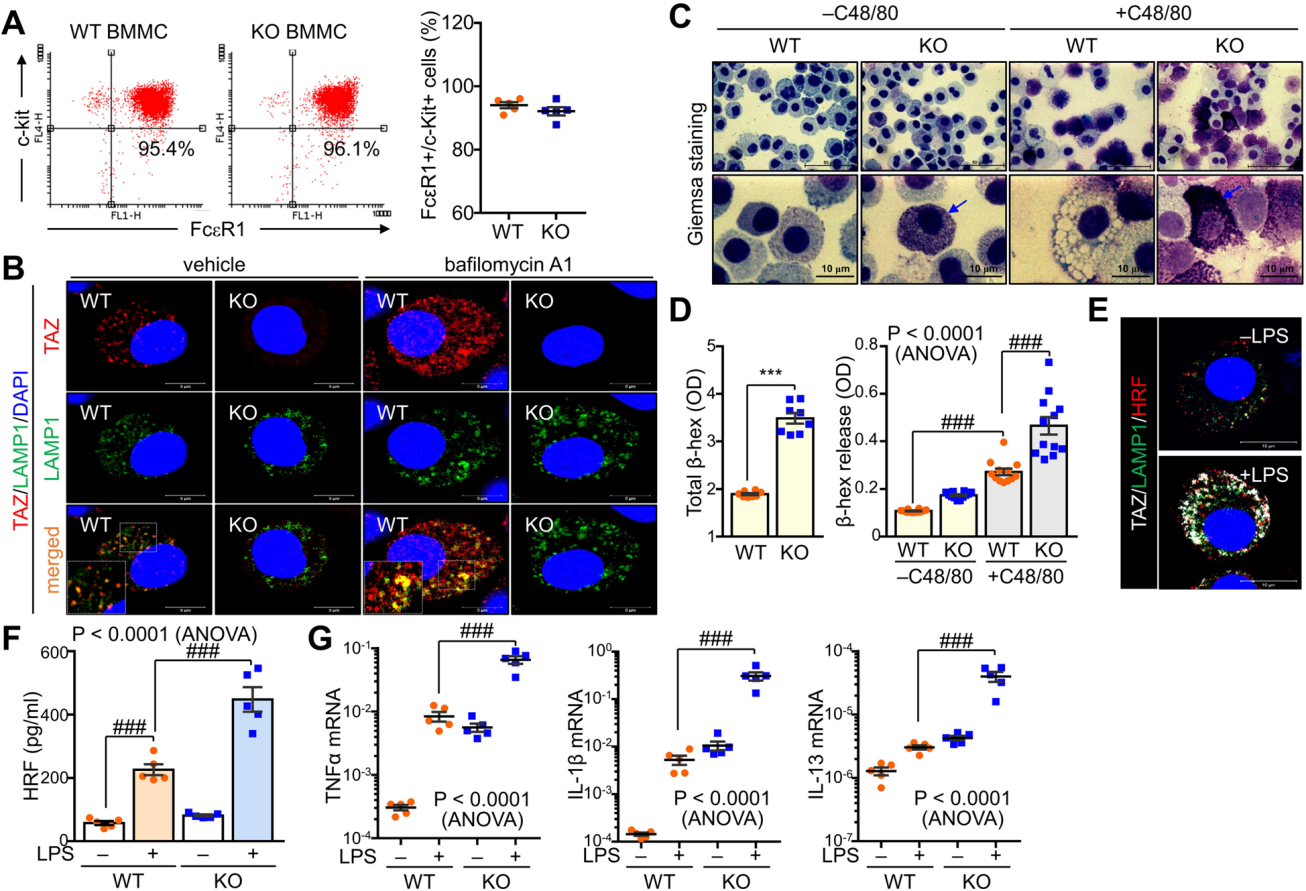
Increased mast cell maturation and degranulation due to TAZ deficiency. BMMCs were established from the bone marrow of WT and TAZ KO mice and cultured in the presence of IL-3 for 35 days. **A** Flow cytometry analysis of BMMCs expressing FcεR1 and c-kit. Data are expressed as the mean ± SEM (n = 5). **B** Immunofluorescence staining for TAZ and LAMP1 in WT and TAZ KO BMMCs. DAPI was used for nuclear staining. **C** Giemsa staining of BMMCs upon C48/80 stimulation. **D** The β-hexosaminidase release assay in WT and TAZ KO BMMCs after treatment with C48/80. Data are expressed as the mean ± SEM (n = 8). ***, *p* < 0.0005 by ANOVA with post-hoc Tukey′s HSD test. **E** BMMCs were stained with antibodies against TAZ, LAMP1, and HRF and subsequently incubated with DAPI, followed by observation with confocal fluorescence microscopy. **F** Culture supernatants of BMMCs stimulated with LPS for 24 h were subjected to HRF ELISA. **G** Total RNA was prepared from LPS-stimulated BMMCs and subjected to determine the relative transcript levels of TNF-α, IL-1β, and IL-13

To investigate the effects of TAZ on mast cell development and activation, we first isolated bone marrow cells from wild-type (WT) and TAZ knockout (KO) mice and generated bone marrow-derived mast cells (BMMCs) in the presence of murine IL-3 for 4 weeks. BMMCs were identified by staining with FcεR1 and c-Kit, and no significant difference in the development of BMMCs was observed between WT and TAZ KO mice (Fig. 1A). We also found that TAZ was expressed in mast cells and co-localized with lysosome-associated membrane protein 1 (LAMP1) in lysosomes, which was further clarified in the presence of the lysosome inhibitor bafilomycin A1 (Fig. 1B). Interestingly, TAZ-deficient BMMCs produced more granules than WT cells and increased mast cell degranulation induced by compound 48/80 (C48/80) (Fig. 1C). Consistently, the total amount of β-hexosaminidase and its release were significantly increased in TAZ KO cells (Fig. 1D). TAZ and HRF in BMMCs were upregulated and co-localized in the lysosomes by stimulation with lipopolysaccharides (LPS) (Fig. 1E). The absence of TAZ enhanced HRF secretion by BMMCs and increased the expression of mast cell cytokines like TNFα, IL-1β, and IL-13 (Fig. 1F), suggesting the potential role of TAZ in regulating BMMCs activity.

### HRF levels are elevated in TAZ-deficient immune cells

**Fig. 2:**
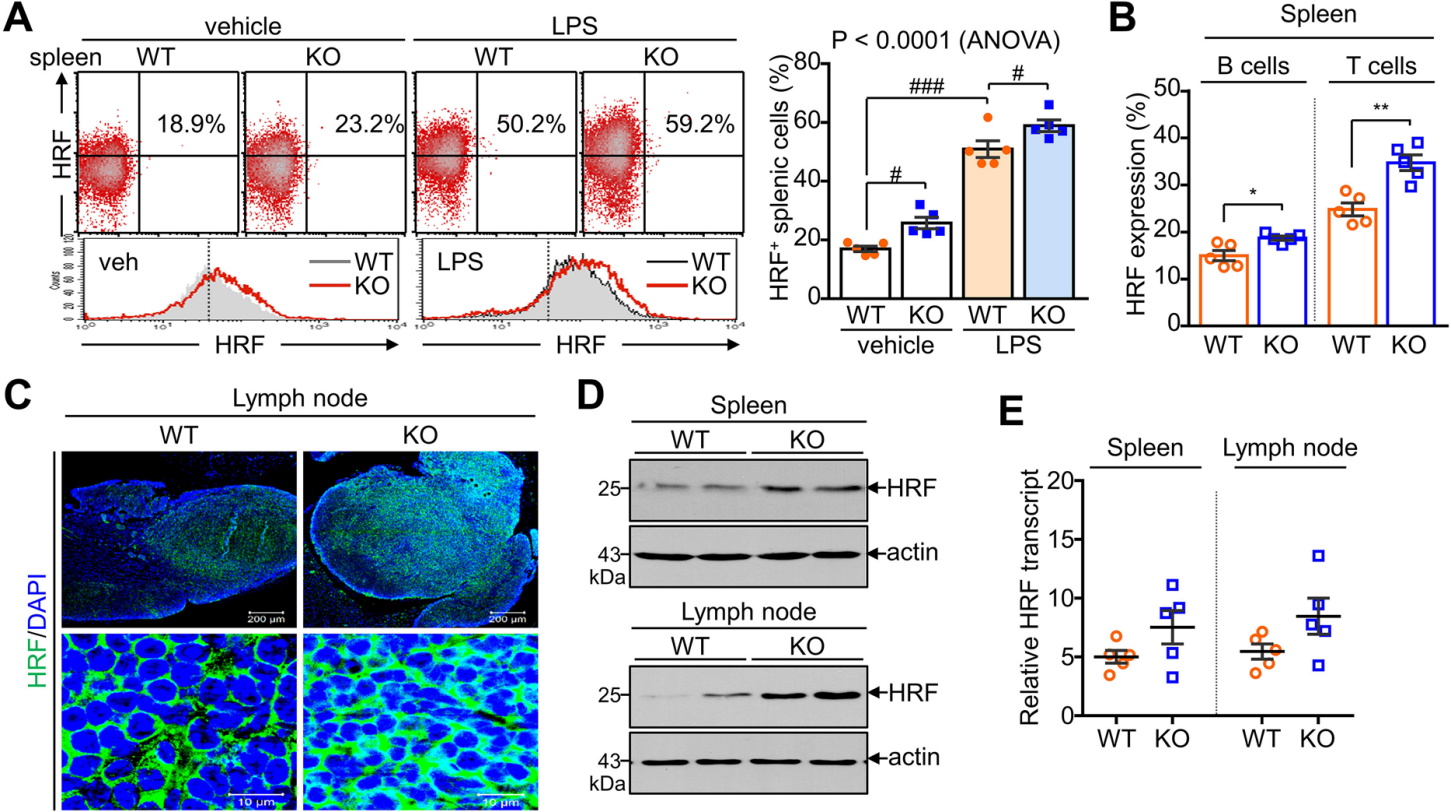
Increased HRF expression in TAZ-deficient immune cells. **A** Single-cell suspensions of the spleens of WT and TAZ KO mice were stimulated with or without LPS (10 ng/mL) for 24 h and stained with anti-HRF antibody, followed by flow cytometry analysis. Data are expressed as the mean ± SEM (n = 5). ^#^*p* < 0.05; ^###^*p* < 0.0005 by ANOVA and Tukey′s HSD test. **B** Spleen cells were harvested from WT and TAZ KO mice and stained with antibodies against HRF, CD4, and B220. HRF expression was quantitatively analyzed in CD4-gated T cells and B220-gated B cells. Data are expressed as the mean ± SEM (n = 5). *p < 0.05 and **p < 0.005 by Student′s *t*-test. **C** The lymph nodes of WT and TAZ KO mice were sectioned and stained with HRF. DAPI was used to stain the nucleus of the cells. **D** Protein lysates were prepared from the spleen and lymph nodes of WT and TAZ KO mice, followed by SDS-PAGE and immunoblotting analysis of HRF and actin. **E** Total RNA was prepared from the spleen and lymph nodes of WT and TAZ KO mice and subjected to quantitative real-time PCR analysis. The relative transcript level of HRF was determined after normalization to the actin level. Data are expressed as the mean ± SEM (n = 5)

Since HRF is a cytokine-like factor that causes mast cell degranulation, we next investigated whether TAZ affects HRF expression in other immune cells. HRF was highly expressed in spleen cells and was further increased by LPS stimulation; moreover, TAZ deficiency substantially increased HRF expression in the spleen (Fig. 2A). Notably, T and B cells, the predominant cell types in the spleen, expressed high HRF levels, which was further promoted by TAZ deficiency (Fig. 2B). Immunohistochemistry analysis confirmed that HRF expression was increased in the lymph nodes of TAZ KO mice compared to that in WT lymph nodes (Fig. 2C). Increased HRF protein expression was further confirmed in the spleen and lymph nodes of TAZ KO mice, as evidenced by immunoblotting analysis (Fig. 2D). However, HRF transcripts, of which levels were much more abundant than actin transcripts in the spleen and lymph nodes, were very similar between WT and TAZ KO mice (Fig. 2E). HRF expression was upregulated at the protein level but not at the transcript level by TAZ deficiency, suggesting that the increase in HRF protein levels in TAZ deficiency may be caused by the regulation of protein stability rather than transcriptional regulation.

### TAZ deficiency increases HRF protein stability by preventing protein degradation

**Fig. 3:**
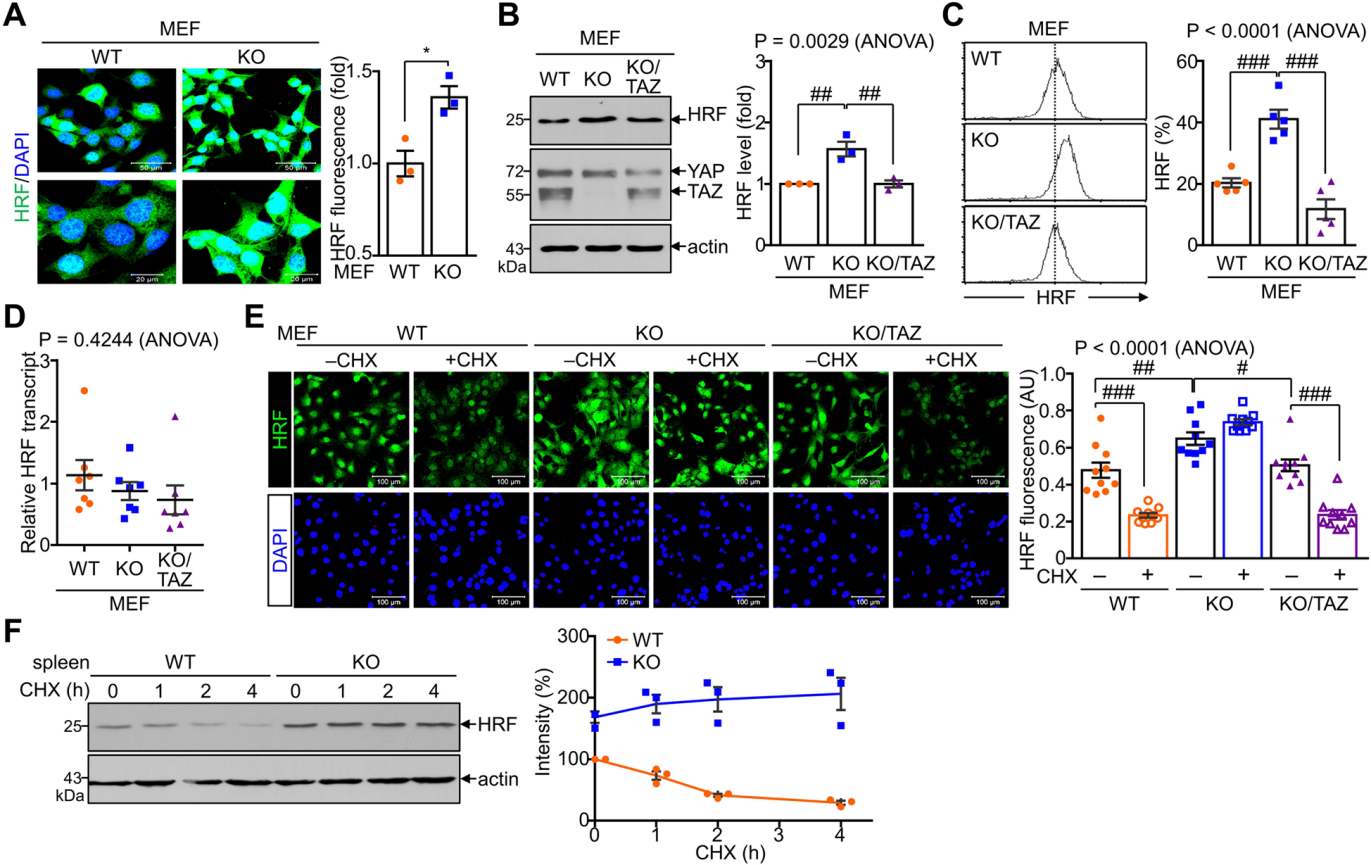
Decreased and increased HRF degradation by TAZ deficiency and restoration. WT and TAZ KO MEFs were obtained from WT and TAZ KO embryos and KO/TAZ MEFs were established by retroviral transduction of TAZ into TAZ KO MEFs. **A** WT and TAZ KO MEFs were stained with anti-HRF antibody and DAPI, followed by confocal fluorescence microscopy. n = 3. *P < 0.05 by Student′s *t*-test. **B** Immunoblot analysis of HRF and TAZ in MEFs. **C** Flow cytometry analysis of HRF in MEFs and quantitative analysis of HRF-expressing cells using the CellQuest software. n = 5. ^###^P < 0.0005 by ANOVA and Tukey′s HSD test. **D** Total RNA was extracted from MEFs and subjected to quantitative real-time PCR to determine the relative HRF transcript. n = 7. The P value was from the ANOVA. **E** MEFs were treated with or without CHX for 6 h and stained with a fluorescence-conjugated HRF and DAPI. HRF intensity in MEFs was quantified in different cell images using the Image J software (n = 10). **F** WT and TAZ KO MEFs were treated with CHX for the indicated time points and subjected to immunoblot analysis of HRF. HRF band intensity was quantified after normalization to the actin level. ^#^*p* < 0.05; ^##^*p* < 0.005; ^###^*p* < 0.0005 by ANOVA and Tukey′s HSD test

Since HRF and TAZ are abundantly expressed in various cell types, we analyzed whether TAZ regulates HRF expression in non-immune cells, such as mouse embryonic fibroblasts (MEF) cells. HRF was expressed in MEF cells and increased in TAZ KO MEFs (Fig. 3A). Increased HRF expression in TAZ KO MEFs was reduced by TAZ restoration, as shown by immunoblotting and flow cytometry (Fig. 3B, C). Consistent with previous findings, HRF transcripts were also abundant in MEF cells and were unaffected by either TAZ deficiency or TAZ restoration (Fig. 3D). Interestingly, HRF protein levels were reduced upon CHX treatment in WT MEFs but not in CHX-treated TAZ KO MEFs. Furthermore, CHX-induced HRF protein degradation in TAZ KO MEFs was rescued by TAZ restoration (Fig. 3E). We found that the 2-h half-life of HRF protein observed in WT cells was extended to more than 4 h in the absence of TAZ (Fig. 3F). These results suggest that TAZ may play an important role in regulating HRF protein stability.

### TAZ is essential for the proteasomal degradation of HRF in spleen cells

**Fig. 4:**
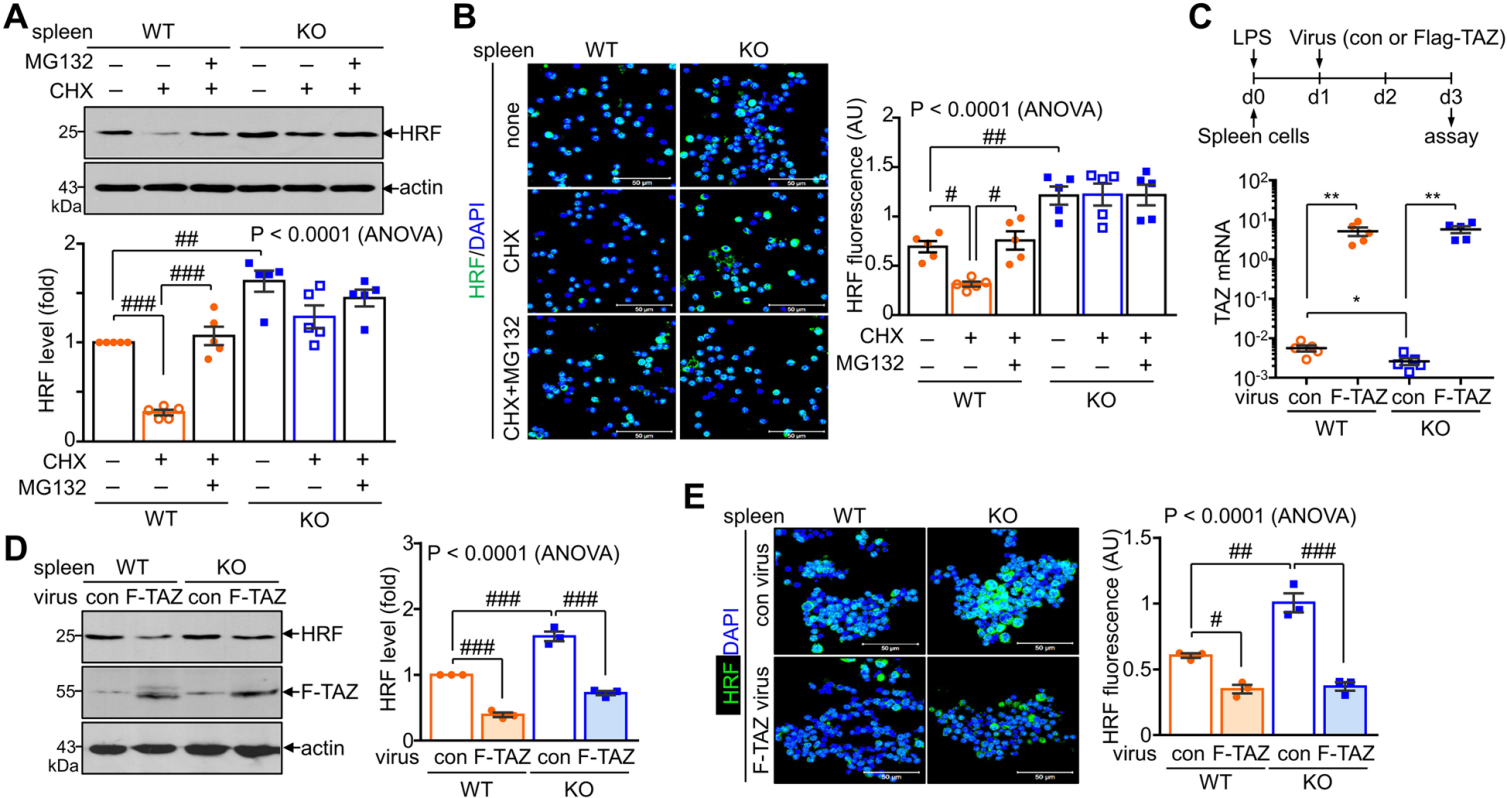
Promotion of proteasomal degradation of HRF by TAZ. Spleen cells were obtained from WT and TAZ KO mice and stimulated with LPS (100 ng/mL). (**A**) Spleen cells were treated with CHX (10 μg/mL) for 6 h in the absence or presence of MG132 (20 μM) and subjected to immunoblot analysis. The intensity of the HRF band was quantified using the Image J software. n = 5. ^##^*p* < 0.005; ^###^*p* < 0.0005 by ANOVA and Tukey′s HSD test. (**B**) Spleen cells were plated in a slide chamber and treated with CHX and MG132 after LPS stimulation, followed by HRF immunofluorescence staining. Fluorescence intensity was quantitatively analyzed using the Image J program (n = 5). ^#^*p* < 0.05 and ^##^*p* < 0.005 by ANOVA and Tukey′s HSD test. (**C-E**) WT and TAZ KO spleen cells were stimulated with LPS and transduced with TAZ by retroviral transduction. Total RNA was collected from virus-transduced cells and subjected to quantitative real-time PCR (n = 5). *p < 0.05; **p < 0.005 by Student′s *t*-test (**C**). Protein extracts were obtained from spleen cells and resolved by SDS-PAGE and immunoblot analysis. n = 3. ^###^*p* < 0.0005 by ANOVA and Tukey′s HSD test (**D**). Viral-transduced spleen cells were plated in a slide chamber and subjected to HRF and DAPI staining. HRF intensity was quantitatively determined after normalization with DAPI intensity. ^#^P < 0.05; ^##^*p* < 0.005; ^###^*p* < 0.0005 by ANOVA with Tukey′s HSD test

The fact that TAZ was required for CHX-induced HRF degradation prompted us to investigate whether TAZ mediates the proteasomal degradation of HRF in spleen cells. HRF expression was reduced by CHX treatment, which was stabilized by the addition of MG132, a proteasomal degradation inhibitor, in WT spleen cells. However, HRF expression was not decreased by CHX in TAZ KO spleen cells and was not significantly affected by MG132 (Fig. 4A). Immunofluorescence staining confirmed that the proteasomal degradation of HRF observed in WT and blocked by MG132 did not occur in TAZ KO cells (Fig. 4B). We then investigated whether TAZ recovery induces the proteasomal degradation of HRF. Retroviral transduction of TAZ into WT and TAZ KO spleen cells comparably increased TAZ expression between WT and TAZ KO cells (Fig. 4C). HRF protein levels were significantly decreased by the ectopic expression of TAZ in both WT and TAZ KO spleen cells, as demonstrated by immunoblotting and immunofluorescence staining (Fig. 4D, E). These results indicate that TAZ is essential for the proteasomal degradation of HRF in spleen cells and that HRF is more abundant in TAZ-deficient cells due to the defects in proteasomal degradation.

### TAZ deficiency increases susceptibility to IMQ-induced psoriasis

**Fig. 5:**
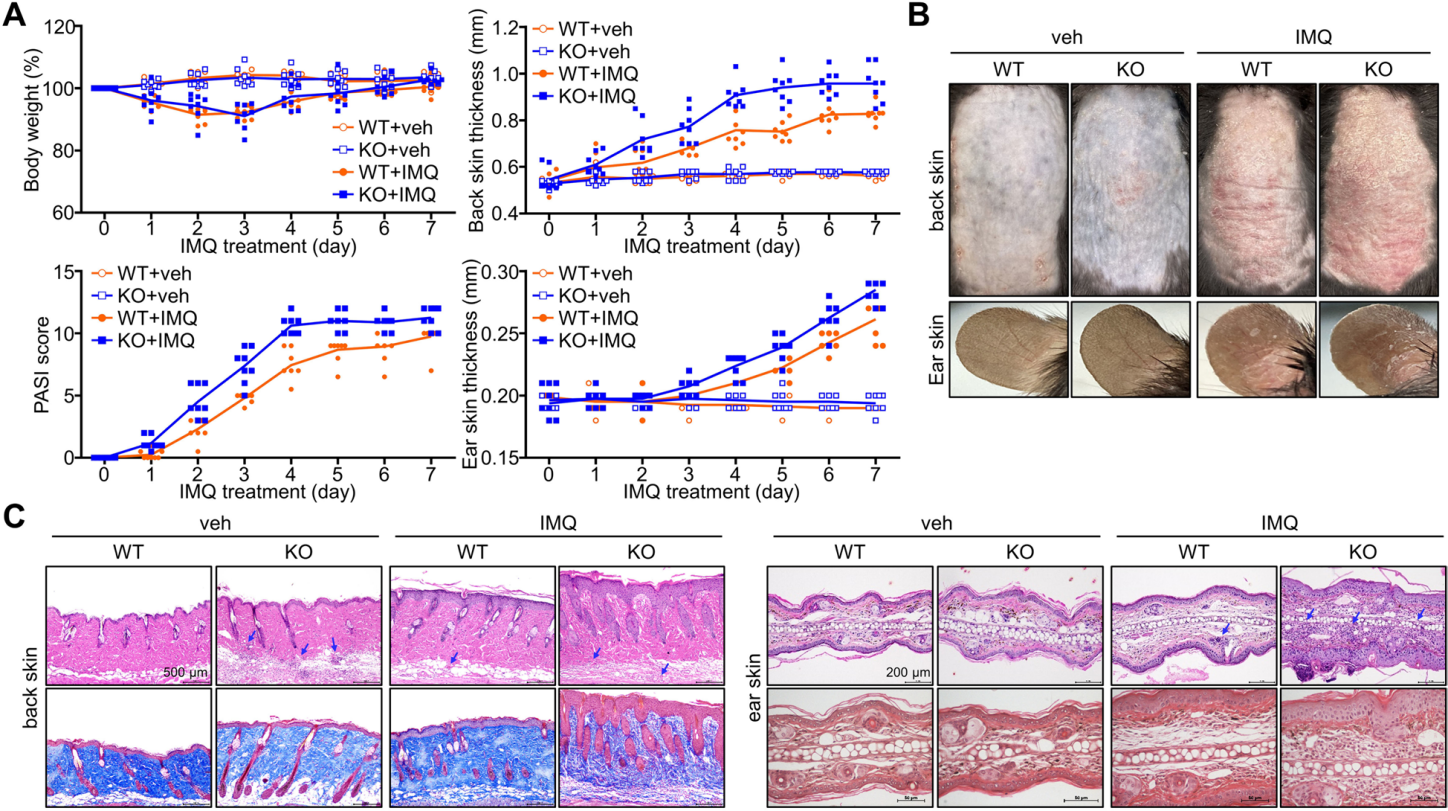
Increased susceptibility to psoriatic inflammation due to TAZ deficiency. WT and TAZ KO mice were treated daily with vehicle or IMQ in the shaved back skin and ear for 7 consecutive days (n = 7–8 per group). **A** Body weight was monitored daily and expressed as a percentage of the weight on day 0. The skin thickness of the back and ear was measured using a dial thickness gauge. The PASI score was determined based on the erythema, scaling, and skin thickness. **B** Photograph of the back and ear skins. **C** Skin tissue sections were stained with H&E and trichrome, followed by microscopic observation. Data are expressed as the mean ± SEM (n = 7 ~ 8). ^#^*p* < 0.05; ^##^*p* < 0.01 by ANOVA and Tukey′s HSD test

Since TAZ deficiency causes HRF protein stabilization and increases mast cell degranulation, we investigated the susceptibility of WT and TAZ KO mice to inflammatory diseases. Topical application of IMQ to the skin of the back and right ear resulted in transient weight loss and gradual weight regain, similar to WT and TAZ KO mice. Notably, IMQ treatment increased the skin thickness of the back and ear in WT and TAZ KO mice. The skin thickness, lesion area, and severity of psoriasis increased in WT mice following IMQ treatment, which was more severely increased in TAZ KO mice (Fig. 5A). Psoriatic lesions with red plaques, silvery scales, and epidermal thickness were induced by IMQ treatment in WT skin and were greatly increased in IMQ-treated TAZ KO skin (Fig. 5B). Histological analysis of the back and ear skin revealed a significant increase in epidermal thickening and immune cell infiltration in WT skin and an even greater increase in TAZ KO skin (Fig. 5C). In addition, immune cell infiltration and fat layer loss were observed in TAZ KO back skin even without IMQ treatment, suggesting that increased HRF expression in TAZ deficiency impairs the maintenance of normal skin tissues and increases susceptibility to psoriatic inflammatory responses.

### TAZ deficiency exacerbates psoriatic pathogenesis with increased HRF expression and secretion

**Fig. 6:**
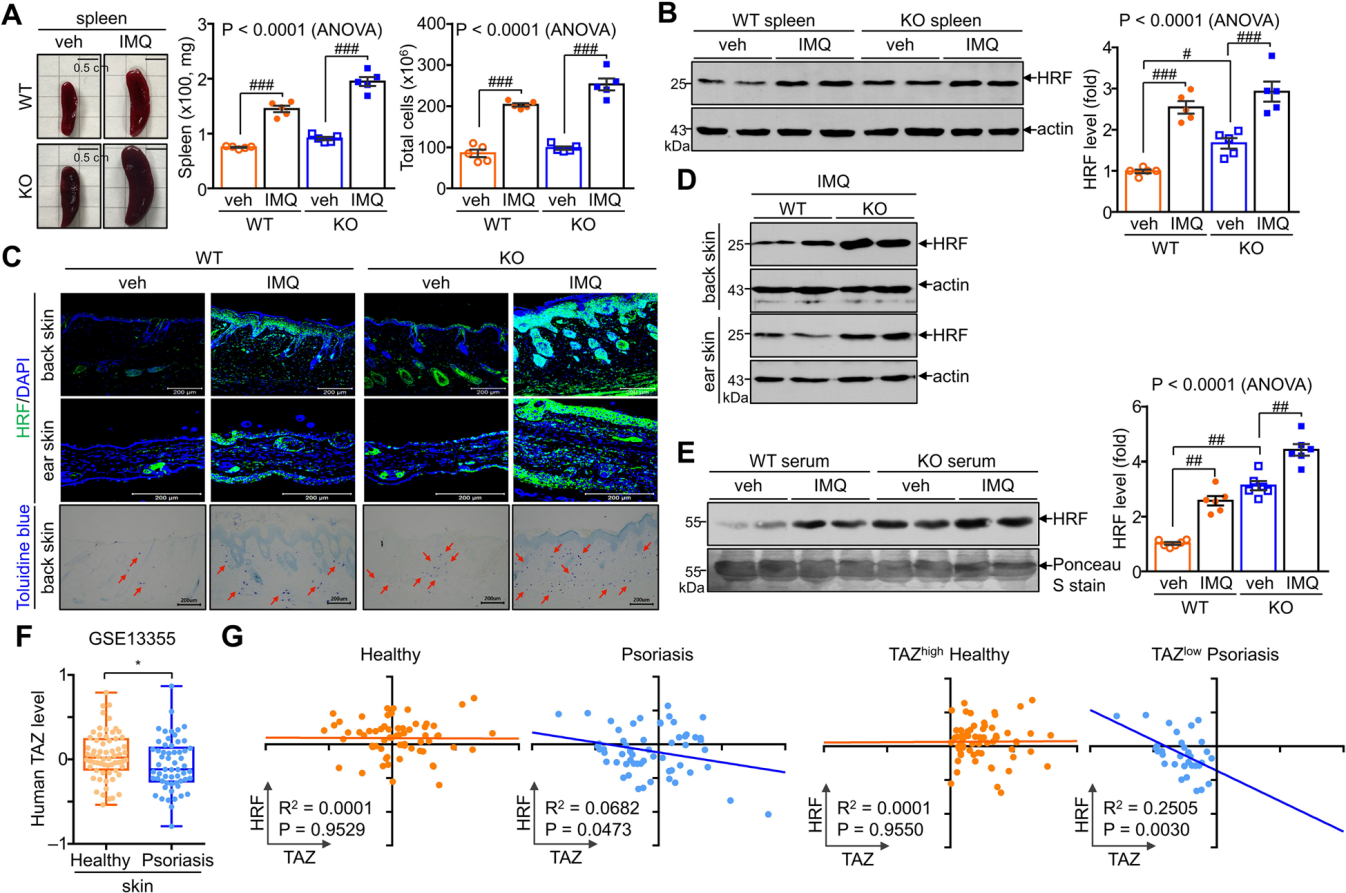
Increased HRF expression and exacerbated psoriasis in TAZ KO mice. WT and TAZ KO mice were treated with vehicle or IMQ in the shaved back skin and ear for 7 days (n = 5 per group). Spleen, dorsal skin, ear skin, and blood samples were collected for further analysis. **A** Size, weight, and cell mass of spleens obtained from WT and TAZ KO mice after treatment with vehicle or IMQ. **B** Immunoblot analysis of HRF in the spleen. HRF levels were quantified by normalization to actin levels and are expressed as a fold change compared to vehicle-treated WT. **C** Skin sections were incubated with fluorescence-labeled anti-HRF antibody or toluidine blue stain, followed by microscopic observation. **D** Protein extracts were collected from the back and ear skin and analyzed by immunoblotting analysis. **E** Serum samples were collected from WT and TAZ KO mice treated with vehicle or IMQ and subjected to SDS-PAGE and immunoblotting analysis of HRF. Extracellular HRF was identified from the size of the dimeric form. Serum immunoglobulins on protein blot were stained with Ponceau S to compare loading levels. **F** Analysis of human TAZ transcripts in the GEO dataset (GSE13355). Normalized TAZ transcript levels were obtained from the skin of normal subjects (n = 64) and the lesional skin of patients with psoriasis (n = 58). **G** Correlation analysis of TAZ and HRF levels in the skin of healthy subjects (n = 58) and patients with psoriasis (n = 58) (top) and TAZ-abundant healthy donor (TAZ^high^ healthy) and TAZ-lacking patients with psoriasis (TAZ^low^ psoriasis) (bottom)

Consistent with the increase in psoriatic pathogenesis in TAZ deficiency, TAZ KO spleens were enlarged compared with WT spleens. Splenomegaly caused by IMQ treatment, which induces a systemic inflammatory response, was more pronounced in TAZ KO mice than in WT mice (Fig. 6A). IMQ treatment increased HRF expression in the spleen, and TAZ deficiency also increased HRF expression in the spleen (Fig. 6B). Increased HRF expression was also found in TAZ KO skin and was significantly increased by IMQ treatment; this increase in HRF levels was highly correlated with mast cell concentration in TAZ KO skin (Fig. 6C). Moreover, immunoblot analysis demonstrated a greater increase in HRF levels in TAZ KO skin than in WT skin (Fig. 6D). In addition, IMQ treatment increased the blood secretion of HRF in WT mice, which was further enhanced by TAZ deficiency (Fig. 6E). Moreover, we found the reverse correlation between TAZ and HRF levels in human patients with psoriasis. The GEO dataset, which analyzed gene expression profiles in healthy donor and lesional skin from patients with psoriasis, showed that TAZ was significantly reduced in the lesional skin of patients compared to healthy subjects (Fig. 6F). And TAZ and HRF levels were inversely correlated in patients with psoriasis, with increased HRF being more pronounced in psoriatic lesional skin with lower TAZ expression, while no correlation was in healthy skin (Fig. 6G). These results suggest that TAZ deficiency exacerbates psoriatic pathogenesis by further promoting HRF production and secretion upon IMQ treatment.

## Discussion

Our results showed that TAZ is expressed in mast cells, particularly in lysosomes, and that TAZ deficiency increases mast cell maturation and degranulation, resulting in a concentration of mast cells in the deep dermis. Furthermore, TAZ deficiency increased HRF expression and its blood secretion due to impaired proteasomal degradation, contributing to mast cell activation and inflammation. Increased HRF levels caused by TAZ deficiency enhanced inflammatory responses and exacerbated IMQ-induced psoriatic inflammation, suggesting the importance of TAZ function in controlling HRF-mediated inflammatory responses and psoriasis development.

TAZ has been shown to promote the development of pro-inflammatory T cells and macrophages and to exacerbate chronic inflammatory diseases [17, 18]. And since TAZ acts very similarly to YAP in the Hippo pathway, we expected it to promote psoriasis development as YAP does in psoriasis [31, 32]. However, we found that TAZ deficiency increased susceptibility to IMQ-induced psoriasis by promoting the production and secretion of HRF, an inflammatory mediator. The unaltered expression of YAP in TAZ deficiency suggests that the exacerbated psoriasis in TAZ KO is not due to increased expression of YAP, but rather a unique function of TAZ in the regulation of HRF stability. HRF exacerbates skin inflammation by stimulating the secretion of inflammatory substances, especially from mast cells in dermal tissue, triggering the infiltration of various immune cells and causing the hyperproliferation and activation of skin epithelial cells. We found that TAZ is expressed in mast cells and that its deficiency increases mast cell maturation and degranulation upon stimulation. Mast cells are derived from the granulocyte/monocyte progenitor populations of the bone marrow and play a key role in allergy and anaphylaxis through the release of various inflammatory mediators, such as histamine, β-hexosaminidase, and HRF [30, 37]. TAZ deficiency did not affect mast cell development from the bone marrow but accelerated mast cell maturation and degranulation. However, TAZ-deficient BMMCs released more inflammatory mediators, including β-hexosaminidase and HRF. HRF protein was intracellularly and extracellularly increased in TAZ deficiency, but HRF transcripts were unaffected by TAZ deficiency. Intracellular HRF mRNA levels appeared to be abundant above the levels of the housekeeping gene actin. Thus, HRF expression may be regulated mainly at the translational level. Indeed, HRF protein levels were decreased in the presence of CHX and inhibited by the addition of MG132, and this proteasomal degradation of HRF was impaired by TAZ deficiency and restored by ectopic TAZ expression in TAZ KO cells. It has been reported that TAZ undergoes proteasomal degradation through interaction with β-transducin repeat-containing protein (β-TrCP) E3 ligase [38, 39], suggesting that TAZ/β-TrCP complex formation may affect the proteolysis of not only TAZ but also HRF, which requires further mechanistic elucidation at a detailed molecular level. In addition, both HRF and TAZ were found to be expressed in lysosomes, indicating the possibility that HRF protein stability may be regulated by altered lysosomal activity in TAZ deficiency, which warrants further investigation.

HRF is an intracellular 25 kDa protein expressed in epithelial cells and various types of immune cells, such as mast cells, B cells, T cells, and macrophages. HRF is secreted extracellularly and undergoes N-terminal proteolysis to form a dimer of approximately 55 kDa. Extracellular HRF reveals multiple immunomodulatory functions, including upregulation of IL-8 production by epithelial cells, modulation of IL-10 production by B cells, promotion of M1 macrophage polarization, inhibition of Treg cells, and activation of Th1 and Th17 cells [25, 30, 35, 40–42]. Increased HRF secretion has been reported in patients with asthma, atopic dermatitis, food hypersensitivity, and psoriasis [25, 35, 43, 44]. We found that TAZ KO mice showed increased HRF expression and exacerbated psoriasis development and that increased TAZ expression promoted HRF protein degradation. Consistent with animal studies, TAZ expression was reduced in human patients with psoriasis, and a correlation has been found between reduced TAZ and increased HRF in psoriasis. Analysis of RNA from punch biopsies taken from the skin of 64 normal healthy volunteers and 58 psoriasis patients confirmed that TAZ transcript was reduced in psoriatic skin samples. However, as the skin of healthy subjects and psoriasis patients show significant differences in the degree of immune cell infiltration, the decrease in TAZ transcript in psoriatic skin samples may be a consequence of increased immune cell infiltration. Nevertheless, while there was no correlation between HRF and TAZ expression in healthy subjects, there was an inverse correlation in psoriatic patients, with low TAZ expression in psoriatic skin being associated with increased HRF. In conclusion, activation of TAZ-mediated HRF proteolysis would be effective and beneficial in regulating various inflammatory diseases, and it would be interesting to develop therapeutics that promote TAZ-mediated HRF proteolysis for controlling psoriasis.

## Conclusions

TAZ deficiency stabilizes HRF expression in immune cells, including mast cells, leading to increased inflammatory responses by HRF. As a result, TAZ deficiency increases susceptibility to psoriatic inflammation caused by IMQ. Thus, TAZ is an important molecule in the control of psoriatic inflammation through the regulation of the pro-inflammatory HRF expression.

## Data Availability

No datasets were generated during this study, and all data are provided in full in the Results section of this paper. Data sets related to this article can be found at https://www.ncbi.nlm.nih.gov/geo/geo2r/?acc=GSE13355) (Tsoi, 2019).

## Abbreviations

BMMCs: Bone marrow-derived mast cells
C48/80: Compound 48/80
CHX: Cycloheximide
DAPI: 4′,6-Diamidino-2-phenyl-indole
HRF: Histamine-releasing factor
IMQ: Imiquimod
KO: Knockout
LAMP1: Lysosomal membrane-associated protein 1
LPS: Lipopolysaccharides
MEFs: Mouse embryonic fibroblasts
TAZ: Transcriptional coregulator with PDZ-binding motif
TCTP: Translationally-controlled tumor protein
WT: Wild-type
